# Acceleration of infectious disease drug discovery and development using a humanized model of drug metabolism

**DOI:** 10.1073/pnas.2315069121

**Published:** 2024-02-05

**Authors:** A. Kenneth MacLeod, Kevin-Sebastien Coquelin, Leticia Huertas, Frederick R. C. Simeons, Jennifer Riley, Patricia Casado, Laura Guijarro, Ruth Casanueva, Laura Frame, Erika G. Pinto, Liam Ferguson, Christina Duncan, Nicole Mutter, Yoko Shishikura, Colin J. Henderson, David Cebrian, C. Roland Wolf, Kevin D. Read

**Affiliations:** ^a^Drug Discovery Unit, Wellcome Centre for Anti-Infectives Research, Division of Biological Chemistry, University of Dundee, Dundee DD1 5EH, United Kingdom; ^b^Division of Systems Medicine, Jacqui Wood Cancer Centre, School of Medicine, University of Dundee, Ninewells Hospital, Dundee DD2 4GD, United Kingdom; ^c^Global Health Research & Development, GlaxoSmithKline, Tres Cantos, Madrid 28760, Spain

**Keywords:** drug discovery, infectious disease, pharmacology, in vivo models, translational research

## Abstract

A key step in drug discovery, common to many disease areas, is preclinical demonstration of efficacy in a mouse model of disease. However, this demonstration and its translation to the clinic can be impeded by mouse-specific pathways of drug metabolism. Here, we show that a mouse line extensively humanized for the cytochrome P450 gene superfamily (“8HUM”) can circumvent these problems. The pharmacokinetics, metabolite profiles, and magnitude of drug-drug interactions of a test set of approved medicines were in much closer alignment with clinical observations than in wild-type mice. Infection with *Mycobacterium tuberculosis*, *Leishmania donovani,* and *Trypanosoma cruzi* was well tolerated in 8HUM, permitting efficacy assessment. During such assessments, mouse-specific metabolic liabilities were bypassed while the impact of clinically relevant active metabolites and DDI on efficacy were well captured. Removal of species differences in metabolism by replacement of wild-type mice with 8HUM therefore reduces compound attrition while improving clinical translation, accelerating drug discovery.

## Significance

Mouse models of disease are widely used to test the efficacy of new compounds during drug discovery and development. However, largely due to species differences in the cytochrome P450 system, mice have a higher capacity for drug metabolism than humans and produce a different spectrum of metabolites. Here, we show that use of mice humanized for these drug-metabolizing enzymes can circumvent these impediments, allowing refocus of compound optimization toward human pharmacokinetics and pharmacodynamics, and improving the alignment of the data generated with clinical observations. The implication of our study is that wild-type mice should be replaced by humanized mice in early and preclinical drug development.

Successful drug discovery and development often relies on the translational accuracy of data generated in animal models. Mice are the animal model of choice across both industry and academia (1–5) due to their ease of husbandry, low cost, short generation time, relative similarity to human physiology and amenability to genetic modification. Demonstration of in vivo efficacy in mouse models of disease is a key early goal when proof of concept (PoC) must be established in an early drug discovery program. This demonstration of PoC is a major step for subsequent optimization efforts involving compound modification, ranking, and go/no-go decision-making. A major barrier to carrying out these studies in mice stems from the fact that a high proportion of compounds shown to be efficacious against the drug target in vitro have poor PK properties in vivo due to high rates of metabolism. Hence, to facilitate demonstration of in vivo efficacy, substantial medicinal chemistry resource must be given to ensuring that new chemical entities (NCEs) are metabolically stable in mice. This chemical remodeling and testing can profoundly increase drug discovery program time and cost, despite the fact that many metabolic liabilities are mouse-specific and ultimately have no bearing on clinical success.

In humans, metabolism is the primary mechanism of elimination for the vast majority of approved small molecule therapies, and 50 to 70% of this metabolism is catalyzed by the cytochrome P450 (CYP) superfamily (6–9). The majority of oxidations associated with drug and foreign metabolism are contained within four CYP multigene families. In humans, there are eight genes within the CYP1A, CYP2C, CYP2D, and CYP3A subfamilies, of which five genes are responsible for over 90% of CYP-mediated drug metabolism in human. In mice, there are 34 genes in these gene families. As a result, there are major species differences between humans and mice in substrate specificity, rates of drug metabolism and in the profile of metabolites produced. Invariably, mice metabolize drugs much more rapidly than humans, which creates a major issue in demonstrating drug efficacy in an early drug discovery program if mouse is the model of disease. In efforts to resolve this issue, several groups, including our own, have “humanized” members of the CYP system through knockout of a mouse gene or gene cluster and replacement, at the same locus, with human gene/s from the equivalent gene subfamily (10–23). However, the utility of these humanized models in translational reseach has been compromised by redundancy in CYP function across subfamilies, with the remaining murine CYPs able to metabolize compounds metabolized by specific CYPs in humans.

To resolve this problem, we have crossed individual humanized mouse lines to create an extensively humanized CYP model, “8HUM” (24). In this model, 33 mouse CYPs from the *Cyp1a*, *2c*, *2d,* and *3a* gene families, together with the transcription factors Pregnane X Receptor (*Pxr*) and Constitutive Androstane Receptor (*Car*) have been deleted and replaced with human *CYP1A1*, *CYP1A2*, *CYP2C9*, *CYP2D6*, *CYP3A4*, *CYP3A7*, *PXR,* and *CAR*. These human enzymes were selected because they are responsible for the vast majority of Phase I metabolism of clinical significance in humans (6–9). We have shown that the levels of hepatic CYP isozyme expression in this model are highly similar to human and that CYP-specific probe substrates are metabolized at similar rates in vitro (24). We have also shown that enzyme levels, for example of CYP3A4, can be regulated by human PXR and CAR activators, and that in vivo DDIs perpetrated by rifampicin (CYP3A4 induction), ketoconazole (CYP3A4 inhibition), quinidine (CYP2D6 inhibition), and sulfaphenazole (CYP2C9 inhibition) all altered the pharmacokinetics of victim drugs in a manner consistent with clinical observations (24). Finally, we have shown that an immunocompromised version of this line, 8HUM_Rag2^−/−^, can be used in xenograft studies (25).

In this report, we describe extensive studies to exemplify the applications of the model in drug development by comparing 8HUM data obtained using a panel of approved medicines with published clinical data. We have evaluated the 8HUM model in an active drug discovery program workflow using industry-standard methodology for the characterization of in vitro microsomal metabolic stability, PK, and metabolite profiling. We demonstrate that, through the use of this in vivo model, the translational relevance of pharmacology is significantly improved. Moreover, in efficacy studies utilizing 8HUM models of infectious disease, we illustrate how this line can be used to bypass mouse-specific metabolic issues that might otherwise halt or substantially delay compound progression.

## Results

### Identification of Compounds for Study.

A set of approved medicines either in use or evaluated for treatment of tuberculosis (TB), Chagas disease, visceral leishmaniasis (VL), malaria, and the common co-morbidity, HIV was identified through queries of the PharmaPendium (Elsevier, Amsterdam, Netherlands) and the University of Washington Drug Interaction Databases (DIDB). Search criteria defined substrates as those compounds for which CYP were listed as a major contributor to metabolic elimination. Inhibitors and inducers are those compounds for which a change to exposure [area under the curve (AUC) or maximum concentration in peripheral blood (C_max_)] of greater than 20% had been reported clinically. We identified multiple drugs for treatment of TB, malaria, and HIV but because of the lack of treatments for kinetoplastid infection, only two for Chagas disease were identified (both of these were triazole inhibitors of CYP51 that failed in the clinic) and none for VL (*SI Appendix*, Table S1).

### Humanization Removes Species Differences in the In Vitro Metabolic Stability of Drugs.

**Fig. 1.**
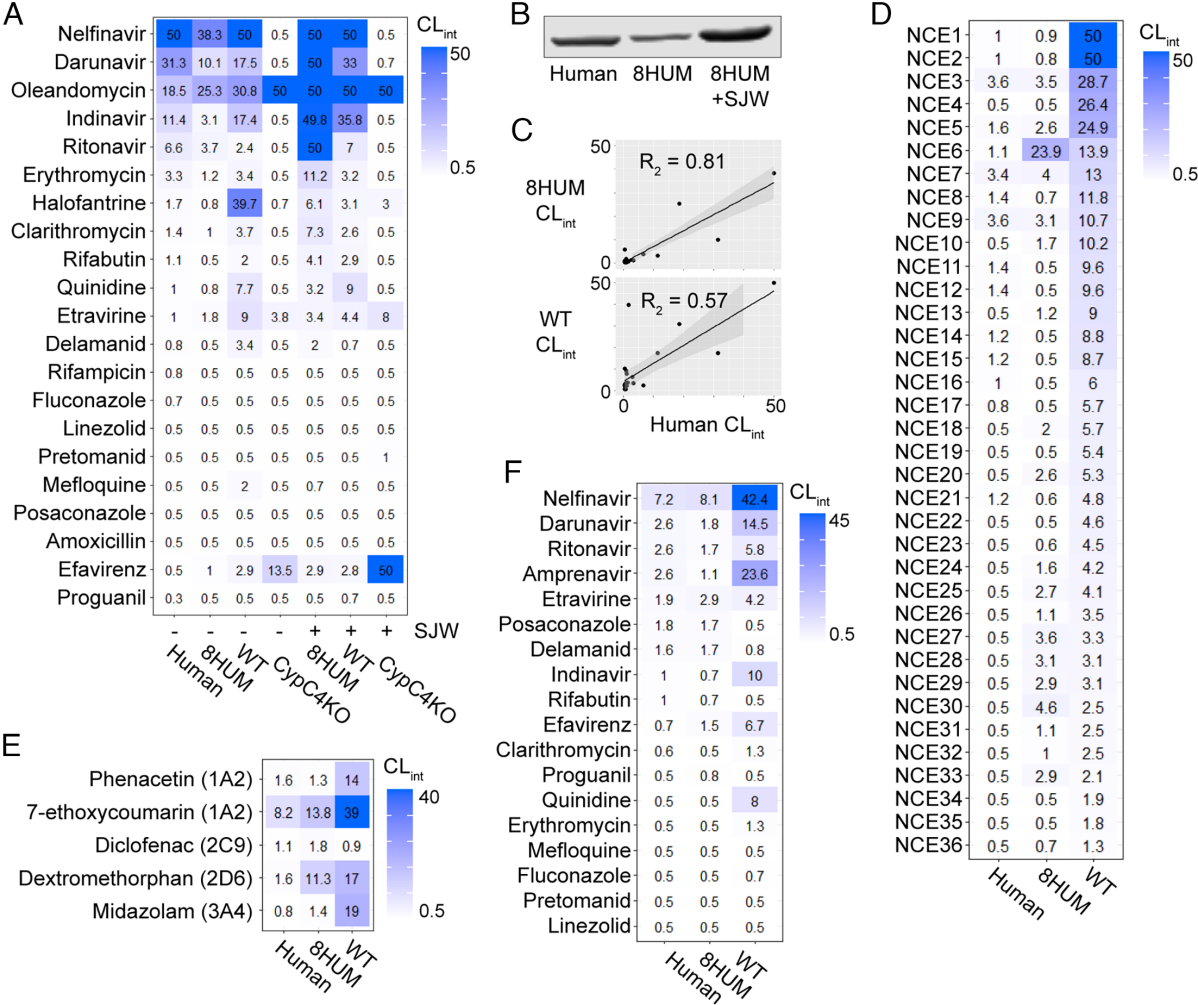
Humanization removes species differences in the metabolic stability of approved medicines and NCEs. (*A*) Heatmap shows intrinsic clearance values of approved medicines in incubations with hepatic microsomes. Microsomes were collected either with (+) or without (−) prior SJW administration. (*B*) Western blot of CYP3A4 protein in liver microsomes from human (pool of 150 individuals), 8HUM (pool of three females), and 8HUM pretreated with SJW (pool of three females). (*C*) Scatter plot of the data for human, 8HUM, and WT shown in (*A*), with linear regression, SE (gray banding), and correlation coefficient. (*D*) Intrinsic clearance of NCEs synthesized at GSK and the University of Dundee Drug Discovery Unit in incubations with hepatic microsomes. (*E*) and (*F*) Intrinsic clearance of CYP-specific probe substrates and approved medicines, respectively, in suspension incubations with cryopreserved (human, WT) and freshly isolated (8HUM) primary hepatocytes. All values are reported as mL/min/g liver, using the scaling factor of 52.5 mg microsomal protein per g liver. Data for some compounds have been omitted due to poor and/or variable MS response.

Intrinsic clearance (CL_int_), a parameter which defines the rate of drug metabolism, of the compounds listed in *SI Appendix*, Table S1 were determined in human, wild-type mouse (WT), and 8HUM hepatic microsomal preparations. An additional microsomal preparation, derived from mice where all the CYPs in the *Cyp1a*, *2c*, *2d*, and *3a* gene families have been deleted from the mouse genome (CypC4KO, Fig. 1*A*) was also included. CYP3A4 is expressed in 8HUM at a lower basal level than in the average human microsomal sample so one group of mice was pre-treated with the CYP3A4 inducer St. John’s wort (SJW). Administration of the Jarsin (Kira) SJW extract, which contains 2.22 to 3.07% of the potent PXR activator, hyperforin (24), resulted in the induction of hepatic CYP3A4 expression to levels higher than in the human liver microsome (HLM) pool (Fig. 1*B*).

As expected for approved medicines, many were metabolically stable across all experimental groups (Fig. 1*A*). Nevertheless, a higher CL_int_ was generally observed in WT than in human, and a much better alignment of the human data with 8HUM was obtained (Fig. 1*C*). In some cases, CL_int_ was higher in 8HUM pre-treated with SJW, which is consistent with the major role CYP3A4 plays in the elimination of many of these compounds in humans (*SI Appendix*, Table S1). With only five exceptions, there was no quantifiable metabolic turnover in CypC4KO microsomes. For four of these compounds, metabolism increased with SJW pre-treatment suggesting that one of the remaining murine CYPs was involved, potentially Cyp2b10, as this protein is inducible in a CAR/PXR-dependent manner^31^. It is noteworthy that for the compounds with higher stability in human liver microsomes (CL_int_ ≤ 2 mL/min/g) and lower stability in WT mouse liver microsomes (CL_int_ ≥ 2 mL/min/g), humanization removed this species difference in almost all cases. To further substantiate this observation, a set of 36 NCEs with the same human/WT difference was assessed in naïve 8HUM microsomes. As for the approved medicines, humanization removed this species difference for the vast majority of compounds (Fig. 1*D*).

Primary hepatocytes are also a key test system for the high throughput assessment of in vitro metabolic stability in early drug discovery. In order to extend our observations to this test system, we implemented a modified two-step collagenase perfusion protocol for the isolation of fresh hepatocytes from 8HUM mice (26, 27). Initial characterization experiments revealed that, for isoform-specific CYP probe substrates recommended by the FDA and EMA, the correlation with human CL_int_ was greatly improved relative to WT mice in 8HUM (Fig. 1*E*). Furthermore, although there were some inconsistencies with the microsomal results [for which there are several potential explanations (28)], this improvement was also seen with the approved medicine set (Fig. 1*F*).

### Translational Relevance of Monotherapy and Combination PK Is Improved in 8HUM.

**Fig. 2.**
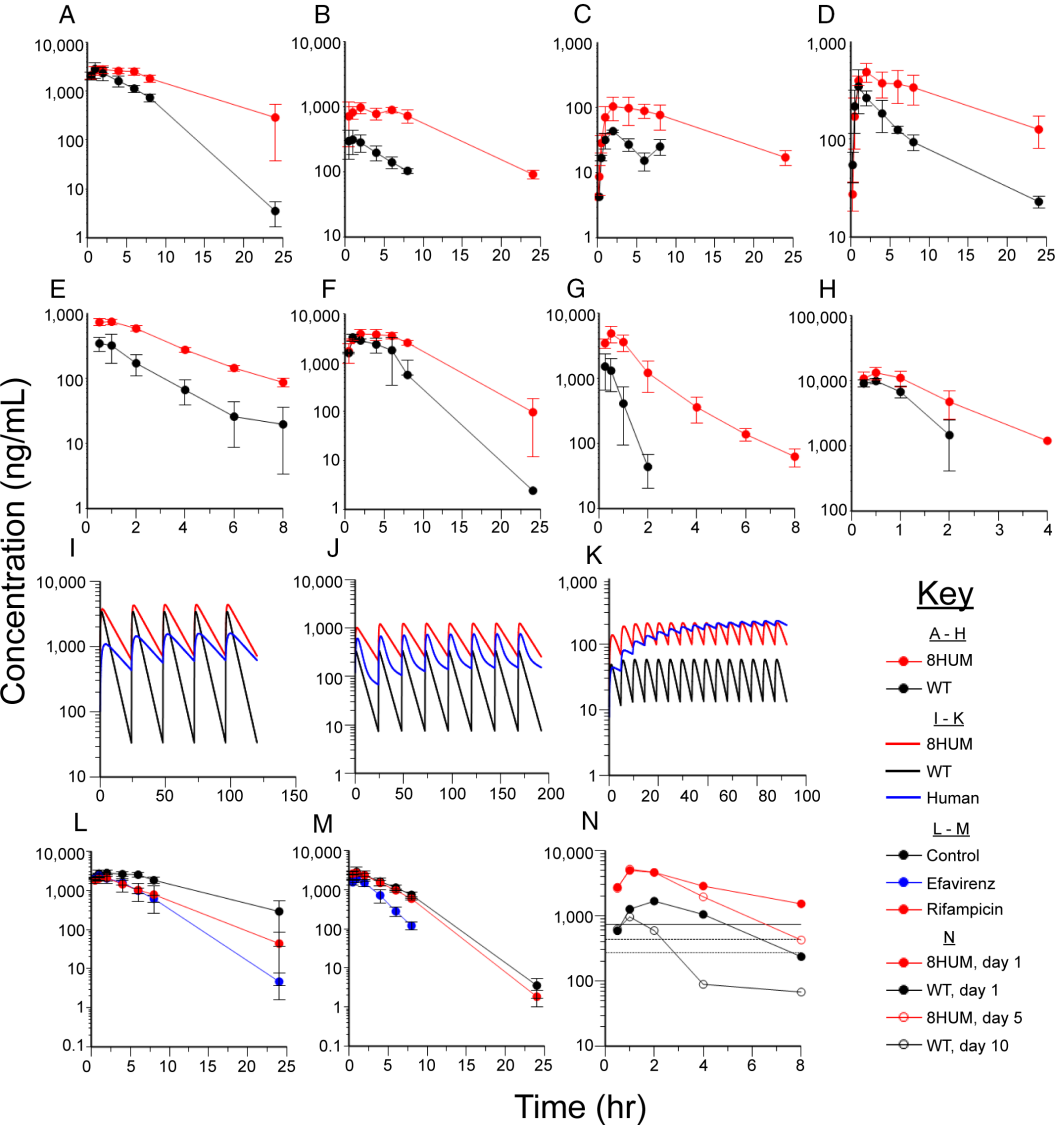
Humanization improves the translational accuracy of monotherapy and combination PK. Concentration versus time plots of total blood compound levels are shown for (*A*) pretomanid, (*B*) rifabutin, (*C*) delamanid, (*D*) bedaquiline, (*E*) quinidine, (*F*) efavirenz, (*G*) NCE37, and (*H*) NCE38 following administration to 8HUM and WT mice. Repeat-dose PK simulations are shown for (*I*) pretomanid, (*J*) rifabutin, and (*K*) delamanid in 8HUM, WT, and human. (*L*) Total blood levels of pretomanid in 8HUM pre-treated with efavirenz, rifampicin, or vehicle. (*M*) Total blood levels of pretomanid in WT pre-treated with efavirenz, rifampicin, or vehicle. (*N*) Unbound levels of NCE39 in 8HUM mice on day 1 or day 5, and in WT mice on day 1 or day 10. Horizontal solid, dashed, and dotted lines represent EC_99_, EC_90_, and EC_50_, respectively. For all observed PK data, n = 3 for each data point with mean ± SD shown.

In order to establish whether pharmacokinetic parameters measured in 8HUM more closely followed those observed in humans, eight compounds were administered orally to WT and 8HUM mice. In all cases, AUC was substantially higher in 8HUM than in WT (Fig. 2 *A–H*). With the exception of pretomanid, C_max_ was also higher. Further, and in line with the microsomal data, apparent oral clearance (CL/F, where F denotes the fraction orally bioavailable) was typically lower in 8HUM than in the WT. For example, for pretomanid, rifabutin, and delamanid, CL/F were 130, 242, and 1,244 mL/h/kg in 8HUM and 266, 1487, and 6,604 mL/h/kg in the WT, respectively. Further studies would be required to determine whether this difference is due to changes in clearance, bioavailability, or both. In order to simulate repeat-dosing PK in mice for comparison with human clinical data, the profiles for three anti-tuberculosis medications were used to generate in silico PK models, as described in *Materials and Methods* (Fig. 2 *I–K*). For all compounds, humanization rendered the PK curve more human-like, primarily due to the “flattening out” effect driven by slower elimination and a consequently lower peak-to-trough ratio.

In order to extend studies to the modeling of DDIs, we investigated the effects of co-administration of CYP3A4 inducers on the PK of pretomanid. In humans, efavirenz has been reported to decrease the AUC and C_max_ of pretomanid by 35% and 28%, respectively (29). At the dose levels studied, which gave clinically relevant levels of exposure, we observed decreases in AUC and C_max_ of 54% and 14% in 8HUM (Fig. 2*L*) and of 65% and 31% in WT (Fig. 2*M*). This interaction was therefore well captured in both genotypes. Notably, efavirenz is an activator of both mouse and human Pxr/PXR (30), and its metabolic elimination may be mediated by the inducible mouse enzyme Cy2b10 (present in both 8HUM and WT), as it is in humans by CYP2B6 (31). In humans, co-administration of rifampicin is reported to decrease the AUC and C_max_ of pretomanid by 66% and 53%, respectively (29). At the dose levels studied, we observed decreases in AUC and C_max_ of 51% and 25% in 8HUM (Fig. 2*L*), and only 10% and 6% in the WT (Fig. 2*M*). The finding that this effect was only observed in 8HUM but not WT is consistent with the high potency of this compound in the activation of human PXR relative to the mouse orthologue (32). This is also consistent with previous observations of the effect of rifampicin on CYP3A4 levels and PK in 8HUM (24).

Murine-specific autoinduction of metabolism leading to reduced exposure can be a major confounder in demonstrating drug efficacy when treatment duration is longer than 3 d. In order to compare this effect in WT and 8HUM mice, we studied an in-house compound from our Chagas disease drug discovery program, NCE37. In WT mice, unbound blood levels of NCE37 were above the predicted target efficacy level (based on an in vitro assay-derived EC_99_) for more than 8 h following administration on day 1 but by day 10 autoinduction had led to unbound blood levels dropping below the target level at approximately 3 h (Fig. 2*N*). In 8HUM, however, we observed a substantial improvement in exposure which was maintained up to 5 d (later timepoints not tested) due to a significantly reduced level of autoinduction, likely a consequence of mouse-specific Pxr activation (Fig. 2*N*). Future studies should compare the same timepoints between genotypes.

### Drug Metabolite Profiles Are Humanized in 8HUM.

Drug metabolites can be pharmacologically active or give rise to toxicity. Species differences in metabolite profiles can significantly affect the translation of drug efficacy and safety studies. To evaluate drug metabolites produced by 8HUM, WT, and human, we studied the metabolite profiles of 14 approved medicines in hepatic microsomal incubation samples. As most of the compounds were CYP3A4 substrates, hepatic microsomes from 8HUM pre-treated with SJW were also included.

**Fig. 3.**
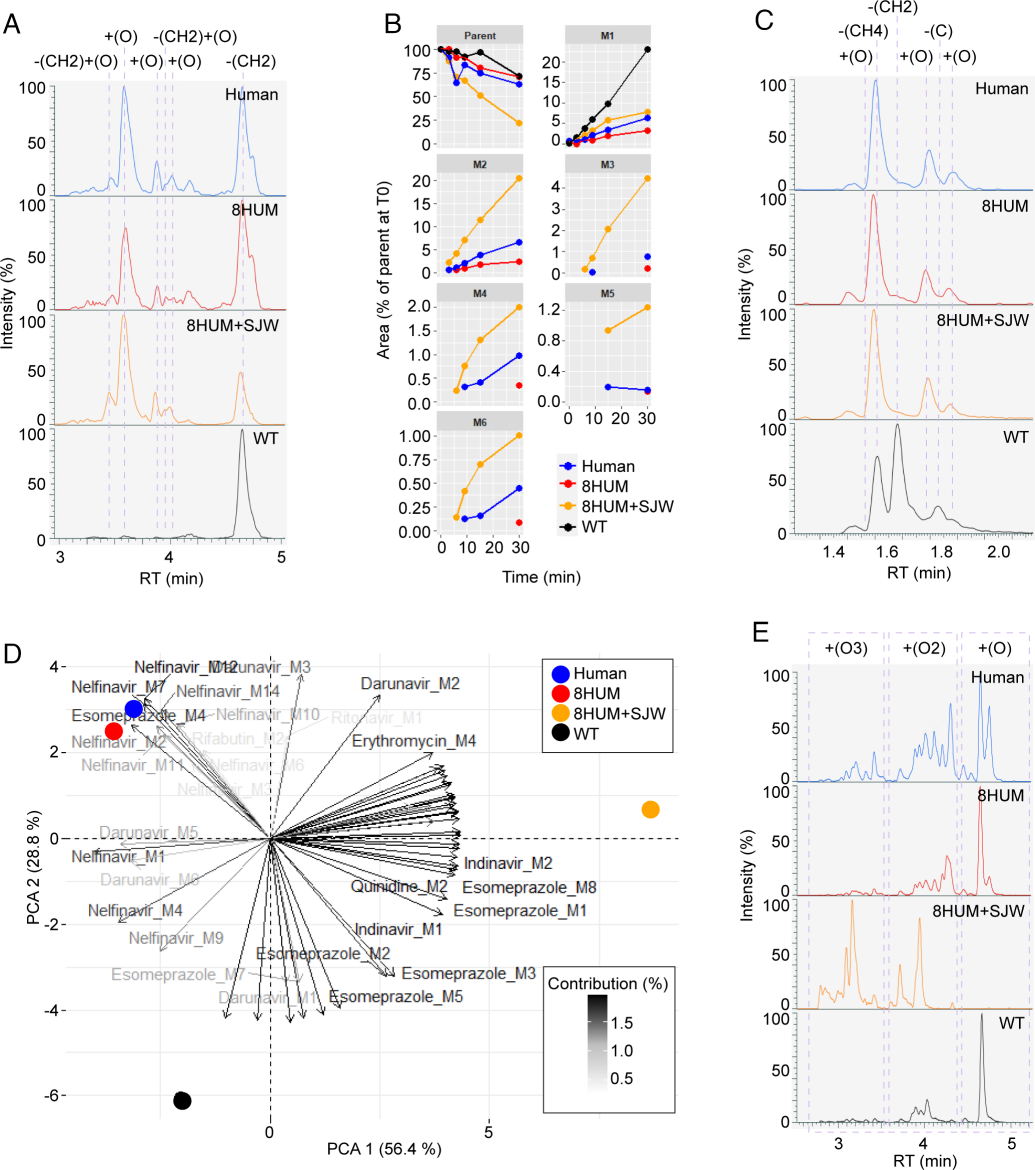
Humanization improves clinical relevance of drug metabolite profiles. (*A*) All-component extracted ion chromatograms (XIC) from LC–MS/MS metabolite profiling analysis of the 30-min samples from incubations of hepatic microsomes with erythromycin. (*B*) Time course of peak areas of erythromycin metabolites in samples from incubations with hepatic microsomes, normalized to the peak area of parent compound at time zero. (*C*) All-component XIC of the 30-min samples from incubations of hepatic microsomes with quinidine. (*D*) Principal components analysis (PCA) biplot showing variables (loadings) derived from normalized metabolite peak areas and their relationship to the test systems (scores). (*E*) All-component XIC of the 30-min samples from incubations of hepatic microsomes with nelfinavir.

Widespread qualitative differences in metabolites produced between the four test systems were observed. In the case of the antibiotic erythromycin, the difference between human and WT mouse was particularly striking. Clinical studies have demonstrated that N-desmethyl (NDM) erythromycin is the major human metabolite (33). This is a product of CYP3A4-mediated metabolism and the basis of the erythromycin breath test, a clinical assessment of an individual’s level of CYP3A4 activity (34). Formation of NDM-erythromycin was observed in all incubations (Fig. 3*A*). However, this was the only metabolite produced in WT mouse samples, whereas in human and 8HUM microsomes five further oxidation products were produced. Indeed, the metabolite profiles of 8HUM and human were almost superimposable. The rate of production of all metabolites increased in 8HUM microsomes following SJW pre-treatment, while the metabolic stability of parent compound greatly decreased, suggesting that CYP3A4 is responsible for all metabolites observed in human and 8HUM (Fig. 3*B*).

CYP3A4 is also the main enzyme involved in the metabolic elimination of quinidine in man, generating 3-hydroxy- and N-oxide metabolites (35). Consistent with this, although we could not assign conclusive identities, the two most abundant metabolites we observed in samples from human and 8HUM incubations involved oxidations of the quinoline and tertiary amine sites of the molecule (Fig. 3*C*). Moreover, we observed a distinct species difference, with O-demethylation (ODM) predominating in mouse. This metabolite was not detected in samples from human or 8HUM. One further oxidation was observed in human, 8HUM+SJW and WT, while two additional metabolites were WT mouse specific: ODM with additional desaturation and loss of the terminal alkene carbon. Hence, for these examples, translational relevance of the in vitro metabolite profile in 8HUM was greatly improved. Further PK and metabolite profiling of 8-aminoquinolines in 8HUM is planned.

In order to summarize the in vitro metabolite profiling results from all 14 compounds studied, we carried out a principal component analysis (PCA) using metabolite peak areas from the final incubation timepoint (30 min) as variables. Human and 8HUM groups clustered closely together, separated by principal component two from the WT group in a clear illustration of the improvement that humanization has on the metabolite profile of the compound set as a whole (Fig. 3*D*). Principal component one described the separation of the 8HUM+SJW group from the others, underscoring the key importance of CYP3A4 to the metabolism of many of the compounds tested. By way of example, nelfinavir was extensively metabolized by human hepatic microsomes, with 18 metabolites detected, although only half of these exceeded 1% of the time zero parent abundance (MS response) at any timepoint (Fig. 3*E*). All putative metabolites of nelfinavir were oxidized forms of the molecule, with single oxidations (n = 6) predominating at later chromatographic retention times, double oxidations (n = 7) eluting earlier, and triple oxidations (n = 5) earlier still. In 8HUM, where CYP3A4 levels are lower than in the average human (Fig. 1*B*), a near-identical set of metabolites was observed, but the distribution of peaks was skewed to the right, with higher relative abundance of single oxidations. In 8HUM+SJW, where CYP3A4 levels are higher than in the average human (Fig. 1*B*), double and triple oxidations predominated. Again, the profile of metabolites generated by WT mouse liver microsomes was qualitatively distinct from those of the other groups.

**Fig. 4.**
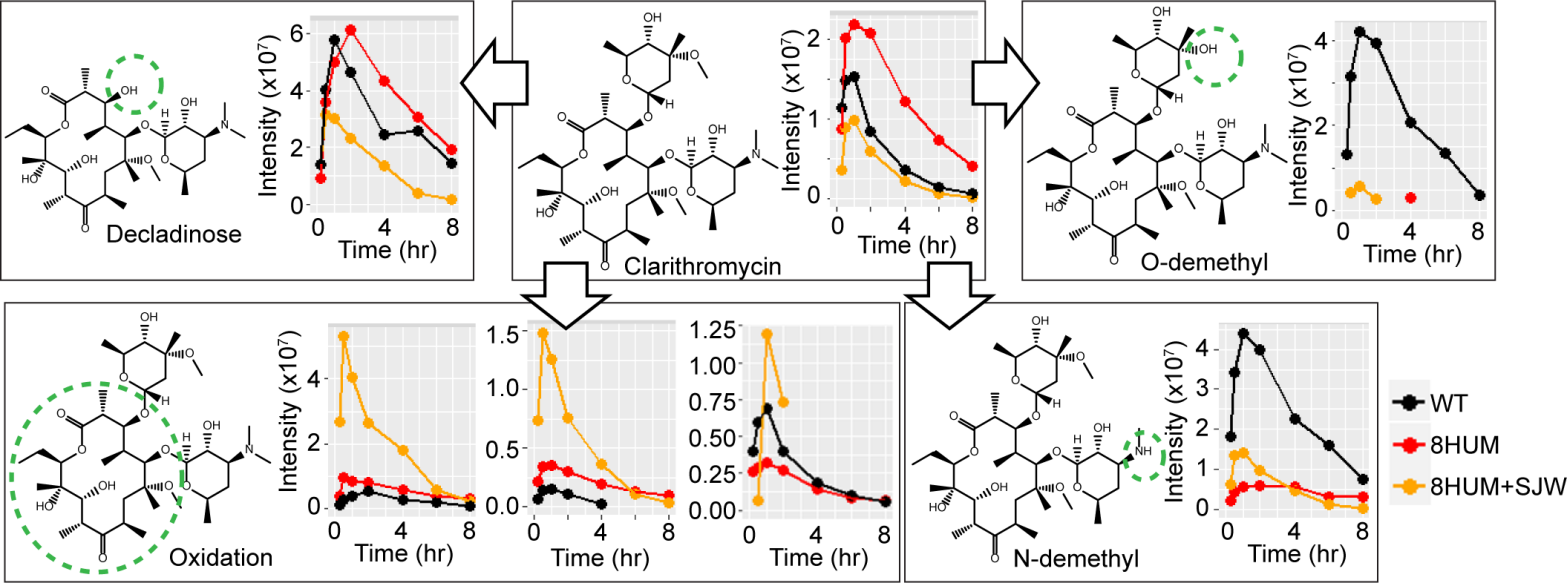
Humanization of in vivo metabolite profile of clarithromycin. Time course of peak areas of clarithromycin and selected putative metabolites in blood samples taken from WT, 8HUM, and 8HUM pre-treated with SJW, following oral administration of clarithromycin at 100 mg/kg (n = 3 in each group, samples from each time point pooled prior to analysis).

To further assess the potential of 8HUM for in vivo generation of key active metabolites, we administered clarithromycin orally to WT, 8HUM, and 8HUM pre-treated with SJW. Clarithromycin is an analogue of erythromycin which differs only in methylation of the 6-carbon hydroxyl, a modification sufficient to improve oral bioavailability, half-life, and tissue penetration (36). In humans, three clarithromycin biotransformation pathways predominate, comprising 14-hydroxylation, N-demethylation, and hydrolysis leading to loss of the cladinose sugar (37, 38). Predominant among these is hydroxylation of the 14-carbon, and it has been reported that all preclinical species, with the exception of the cynomolgus monkey, produce negligible amounts of this key active metabolite (37, 38). In blood samples taken from mice dosed orally, we observed three oxidations of the lactone ring (Fig. 4). Two of these were closely matched in chromatographic retention time (4.25 and 4.30 min) and present in greater abundance in 8HUM than in WT mice. Moreover, this abundance increased substantially with SJW pre-treatment. As 14-hydroxy clarithromycin is a product of CYP3A4-mediated metabolism (39, 40), it is likely that these putative metabolites are its R- and S- epimers, although further analysis with authentic standards is required. Levels of the third oxidation were more closely aligned between all three experimental groups, as were those of a metabolite corresponding in mass to the decladinose form. We observed that NDM-clarithromycin, also formed by CYP3A4 (39, 40), was more abundant in samples from 8HUM following pre-treatment with SJW but was also eliminated more rapidly, suggesting secondary metabolism of this compound. In a key species difference, however, levels of both NDM- and ODM-clarithromycin were far higher in WT than in either of the 8HUM groups. Collectively, these results demonstrate that the translational relevance of the in vivo metabolite profile of clarithromycin is greatly improved with humanization.

### Validation of an 8HUM Model of Acute Tuberculosis Infection and Improved Modeling of the Impact of DDI and Active Metabolites on Efficacy.

**Fig. 5.**
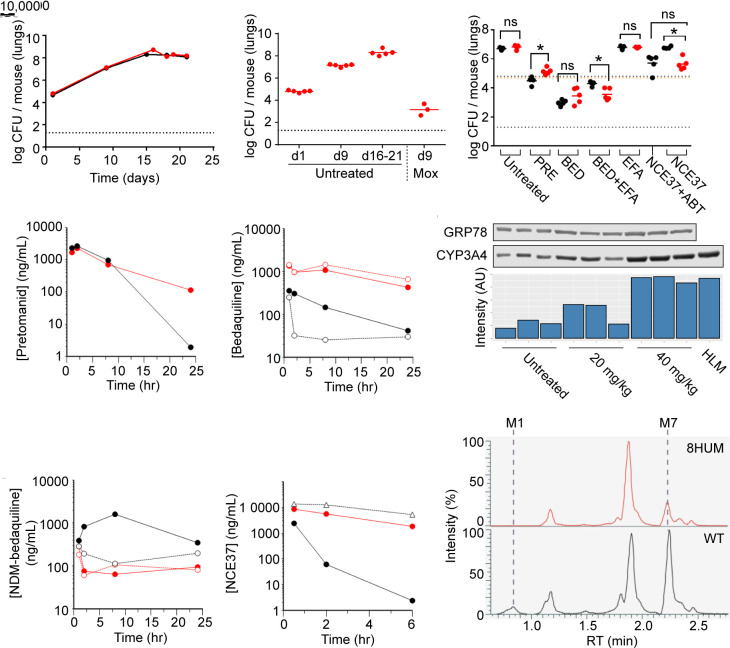
Clinically relevant DDI and active metabolite levels are captured within compound efficacy assessments in an 8HUM model of tuberculosis infection. (*A*) 8HUM (red) and WT (black) mice were intratracheally infected with *M. tuberculosis* and lungs collected at the indicated timepoints for assessment of bacterial burden in lung tissue by CFU assay. (*B*) The effect of moxifloxacin treatment (30 mg/kg QD) on lung CFU assay results in 8HUM infected with M. tuberculosis. (*C*) The effect of pretomanid, bedaquiline, and NCE37 treatment on lung CFU assay results in 8HUM (red) and WT (black) mice infected with *M. tuberculosis*. Blood levels of (*D*) pretomanid and (*E*) bedaquiline in 8HUM and WT were assessed on the final day of dosing (8HUM: filled red circles, WT: filled black circles, 8HUM+efavirenz: open red circles, WT+efavirenz: open black circles, WT+ABT: open black triangles). (*F*) Western blot and chemiluminescence signal intensity showing the effect of 20 and 40 mg/kg QD efavirenz on the hepatic level of CYP3A4 in 8HUM. Blood levels of (*G*) N-desmethyl bedaquiline and (*H*) NCE37 in 8HUM and WT were assessed on the final day of dosing (symbols as for (*D*) and (*E*)). (*I*) All-component XIC for putative metabolites of NCE37 in blood samples from 8HUM (red) and WT (black) taken 1 h after compound administration.

We carried out a pilot head-to-head infection study to assess whether 8HUM can be used in place of WT mice in a model of acute *Mycobacterium tuberculosis* infection (41) The course of infection in 8HUM matched that of the WT strain typically used (C57BL/6J), with a log colony forming unit (CFU) assay increase of 2.3 from days 1 to 9 and a plateau by day 16 at 8.3 log CFU (Fig. 5*A*). No difference in bodyweight between groups was observed. In parallel, we evaluated the ability of our standard positive control, moxifloxacin, to inhibit growth of *M. tuberculosis*. On day 9, a 3.9 log CFU reduction in the lungs compared to the control group was measured (Fig. 5*B*). This was within the standard acceptance criterion of previous studies in WT mice (mean ± 2 SDs) so 8HUM therefore fulfilled the criteria for efficacy evaluation of acute phase *M. tuberculosis* drugs.

Pretomanid is among the new generation of drugs approved for treatment of multidrug-resistant tuberculosis (MDR-TB) (42). The activity of pretomanid is time dependent (43) and, as our single-dose PK study revealed longer half-life in 8HUM than WT, we anticipated that this compound would be more efficacious in 8HUM due to more sustained exposure above the minimum inhibitory concentration of 60 ng/mL (43). However, no such difference was observed (Fig. 5*C*), and in fact, pretomanid was less effective in 8HUM. In part, this was explained by the fact that PK analysis after the day 8 dose (Fig. 5*D*) suggested that the difference between 8HUM and WT at steady state was not as pronounced as had been observed with a single dose (Fig. 5*D*).

Patients with drug-resistant MDR-TB are also treated with bedaquiline. Bedaquiline efficacy is partially mediated by an active metabolite, NDM-bedaquiline although, in patients at steady-state, the AUC ratio of bedaquiline to NDM-bedaquiline is approximately 1: 0.25, and the metabolite is approximately fivefold less potent than the parent compound (44). Efficacy of bedaquiline is dependent on overall exposure (AUC); however, translation of this relationship from dose fractionation studies carried out in murine infection models to the clinic is confounded by the fact that mice produce much more of the active metabolite (44). In 8HUM, we observed an AUC_0–24_ ratio of bedaquiline to NDM-bedaquiline of approximately 1:1 (19,105:19,936 h*ng/mL), much closer to the clinical situation than the 1:72 (2,933:212,227 h*ng/mL) we observed in WT mice (Fig. 5*G*). Hence, the contribution of this active metabolite to inhibition of *M. tuberculosis* growth is modeled more appropriately within this efficacy study. Moreover, this large excess of metabolite may explain why no difference was observed in efficacy between 8HUM and WT treated with the same dose of bedaquiline, despite C_max_ and AUC_0-24_ being 4.4-fold and 6.5-fold higher, respectively, in 8HUM.

Patients with TB often present with HIV as a comorbidity, which may be treated with efavirenz. Efavirenz is an inducer of CYP3A4, the enzyme which catalyzes N-demethylation of bedquiline, but the likelihood of a clinically significant DDI between these compounds is considered low because at steady-state efavirenz decreases the AUC of a single dose of bedaquiline by only 20% and has negligible effect on C_max_ (45). However, in silico simulations have suggested that, under steady-state conditions for both medicines, efavirenz may reduce exposure to both bedaquiline and N-desmethyl bedaquiline by up to 50% (46). This discrepancy between the acute and steady-state situations arises due to the extremely long terminal half-life of bedaquiline [5.5 mo (45)], and illustrates the difficulties inherent in predicting long-term DDI potential in a short-term study format. We therefore carried out a DDI study with efavirenz in 8HUM. This compound had little impact on the PK of bedaquiline and NDM-bedaquiline (Fig. 5 *E* and *G*), and no impact on its efficacy (Fig. 5*C*), despite both compounds being present at levels reported clinically (44, 47), and a robust, approximately threefold, induction of hepatic CYP3A4 (Fig. 5*F*). In WT mice, however, there was a 1.3 log CFU decrease in efficacy (Fig. 5*C*), commensurate with a decrease in bedaquiline AUC of 71% and a PK curve shape that suggested an increased rate of elimination prior to attainment of distribution equilibrium (Fig. 5*E*). The observations in 8HUM, unlike the WT data, were therefore consistent with the clinical consensus that this DDI is not significant.

### 8HUM Removes the Need to Co-Administer the Pan-CYP Inhibitor, 1-Aminobenzotriazole, in Efficacy Studies.

The preclinical efficacy of compounds with high CYP-mediated metabolism is routinely investigated with co-administration of the mechanism-based pan-CYP inhibitor, 1-aminobenzotriazole [ABT (48)]. This approach has its limitations, however, due to potential interference with the efficacy and safety profiles of the test compound, and the increased stress associated with additional dosing steps. Due to rapid CYP-mediated metabolic elimination of NCE37, previous *M. tuberculosis* studies in WT mice have involved ABT co-administration. In 8HUM, however, the levels of NCE37 were substantially increased without the need of this PK-enhancing co-therapy (Fig. 2*G*). We therefore investigated whether this was sufficient to achieve efficacy. Consistent with the previous studies, co-administration of ABT was required for efficacy of NCE37 in WT mice (Fig. 5*C*), and was commensurate with increased drug exposure (Fig. 5*H*). In 8HUM, however, a similar efficacy was observed without the requirement for ABT co-administration (Fig. 5*C*). To identify metabolic routes that could explain the species differences in metabolism of NCE37, we profiled metabolites in blood samples from 8HUM and WT. Of the seven putative metabolites identified, two were observed at lower levels in samples from 8HUM (Fig. 5*I*).

### Use of 8HUM to Bypass Mouse-Specific Metabolic Liabilities in a Model of Kinetoplastid Infection.

To assess whether 8HUM could be used in kinetoplastid drug discovery, we studied the course of infection with *Leishmania donovani* and *Trypanosoma cruzi*, the parasite agents of visceral leishmaniasis (kala-azar) and Chagas disease, respectively.

**Fig. 6.**
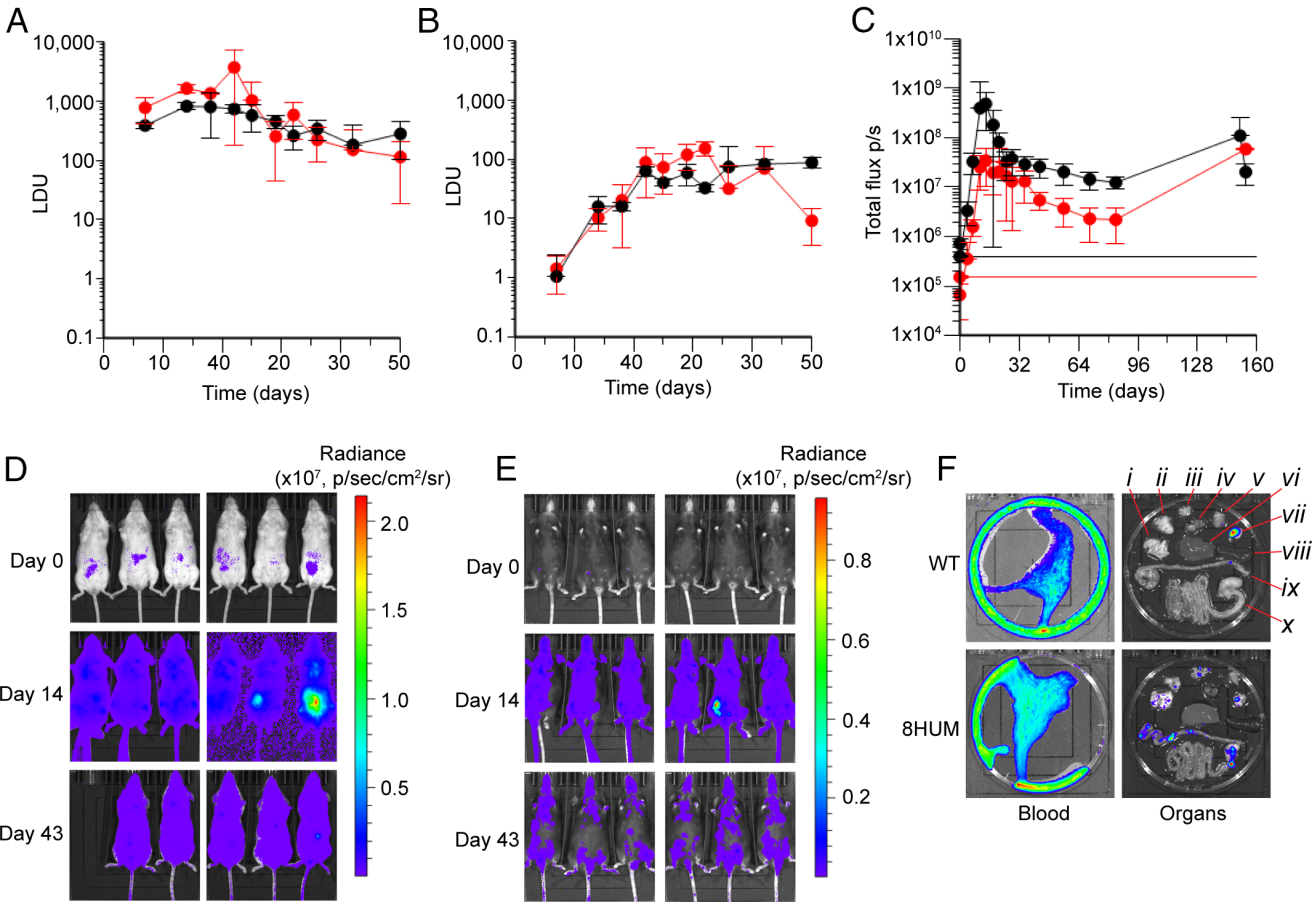
8HUM models of kinetoplastid infection are valid for assessment of compound efficacy. Following infection with *L. donovani*, timecourses of parasitaemia are shown for 8HUM and WT (Balb/c) (*A*) liver and (*B*) spleen. The number of amastigotes per 500 liver cells was counted microscopically (10× 100, oil immersion) and the parasite load expressed in Leishman Donovan Units (LDU), calculated by multiplying the mean number of amastigotes per cell by the tissue weight in mg (49, 50) (n = 2 for each data point, mean ± SD shown). (*C*) Timecourse of summed whole-body bioluminescence signal (dorsal + ventral) from in vivo imaging of 8HUM and WT (Balb/c) following infection with *T. cruzi* CL Brener-Luc strain (51) (n = 5 to 6 for each data point, mean ± SD shown). Horizontal red and black lines denote background signal intensity for 8HUM, and WT, respectively. (*D*) and (*E*) Ventral images for WT and 8HUM, respectively, showing bioluminescence on day 0 (immediately after infection), day 14 and day 43. (*F*) Ex vivo imaging of tissues from (*C*), carried out on day 154 of the experiment. Tissues are identified as follows: (i) lung, (ii) gut mesenteric tissue, (iii) lymph nodes, (iv) heart, (v) skeletal muscle, (vi) liver, (vii) visceral fat, (viii) spleen, (ix) colon and cecum, and (x) small intestine and stomach.

Following infection with *L. donovani*, the temporal course of parasitaemia in both liver (Fig. 6*A*) and spleen (Fig. 6*B*) in 8HUM closely matched that of the WT strain routinely used (Balb/c). However, there was no difference in experimental severity or mortality in 8HUM demonstrating it to be a viable model for evaluation of inhibitors of *L. donovani* growth.

Following infection with a luciferase-labeled strain of *T. cruzi* (51), in vivo imaging of infectious burden revealed that the temporal course of parasitaemia in 8HUM matched that of WT (Balb/c), although bioluminescence signal intensity was lower (Fig. 6*C*). As the fur of Balb/c mice is white, and that of 8HUM (C57BL/6NTac substrain background) is black, this discrepancy was considered likely due to the black fur of the latter inhibiting transmission of the bioluminescence signal (Fig. 6 *D* and *E*). Infection was evident in multiple tissues of 8HUM during ex vivo analysis (Fig. 6*F*). This 8HUM model of infection was therefore considered viable for evaluation of inhibitors of *T. cruzi* growth.

**Fig. 7.**
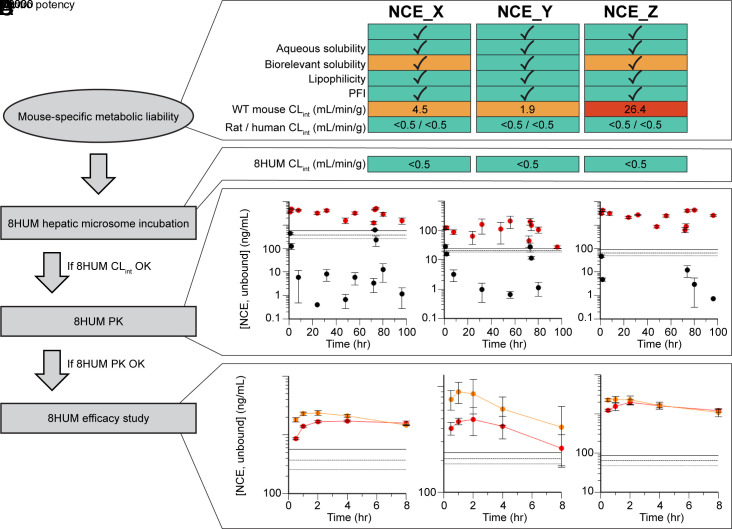
Implementation of 8HUM in Chagas disease drug discovery cascade. (*A*) example data for three compounds for which in vitro and physicochemical properties were successfully optimized, but for which metabolic instability in WT mice (CL_int_, hepatic microsomes) was a persistent problem. (*B*) In vitro stability was greatly improved in 8HUM. (*C*) This translated to an improvement in PK parameters in vivo, with unbound drug levels in 8HUM (red) sustained well above those in WT (black). Solid, dashed, and dotted horizontal lines represent EC_99_, EC_90_, and EC_50_, respectively. n = 3 for each data point with mean ± SD shown. (*D*) In the 8HUM T. cruzi infection model, high unbound drug levels were sustained from the beginning (day 135, red) to the end (day 154, orange) of the treatment window. Horizontal solid, dashed, and dotted lines represent EC_99_, EC_90_, and EC_50_, respectively. n = 4 to 6 for each data point with mean ± SD shown.

The University of Dundee Drug Discovery Unit and GSK Tres Cantos have a long-standing partnership in Chagas disease drug discovery. As part of this fully integrated collaboration, substantive medicinal chemistry resource has been expended in optimizing key properties of a series of compounds with promising in vitro activity (Fig. 7). However, despite favorable in vitro metabolic stability in incubations with hepatic microsomes from human and rat, species-specific metabolic instability with microsomes from mouse has often been a block to compound progression. Following the validation work described above, we are now integrating 8HUM within this project to circumvent this issue. The general workflow describing implementation is shown in Fig. 7, alongside example data for three of the lead compounds. Consistent with the data showing removal of species difference in CL_int_ through humanization (Fig. 1), in vitro metabolic stability was greatly improved for all three compounds, and all three were therefore progressed to PK study in 8HUM. Following our standardized approach of administration at 50 mg/kg BID for 4 d, we observed free drug concentrations above the EC_99_ at all sampling timepoints. This was in stark contrast to WT mice where the metabolic instability previously observed in vitro translated to extremely poor compound exposure in vivo. Subsequently, all three compounds were evaluated for efficacy in the 8HUM *T. cruzi* infection model. Serial blood sampling on days 135 and 154 (the first and last days of treatment) indicated that target drug free concentrations were achieved for the duration of sampling. This improved PK coincided with full cures for NCE_Y and NCE_Z (six of six and five of five mice, respectively) and a partial cure for NCE_X (four of five mice).

## Discussion

Compounds will only usually be considered for candidate nomination in drug discovery programs if they demonstrate efficacy in a validated preclinical disease model. A predominant limitation of these models results from differences in drug metabolism. Here, through validation studies in active drug discovery workflows for infectious disease, we have demonstrated that the use of the 8HUM mouse line, the most complex genetically humanized line to date, can resolve this problem.

We have shown that, for the majority of compounds with a species difference in in vitro metabolic stability, this difference was resolved in 8HUM. In vivo PK analysis substantiated the in vitro findings: AUC and C_max_ were higher in 8HUM than in WT, while apparent oral clearance was lower. Collectively, these data exemplify a key advantage of 8HUM, i.e., the removal of high activity mouse enzymes that limit exposure to candidate compounds in murine models of disease. This will be of great value in early drug discovery PoC studies as it will allow efficacious drug exposure to be reached more easily and go/no-go decisions to be made earlier, thereby avoiding the need for medicinal chemistry modifications targeted purely at resolving mouse-specific metabolism. Although co-administration of the pan-CYP inhibitor, ABT, is commonly used to boost exposure to compounds with high mouse clearance, this approach increases the complexity of the experimental protocol—to a degree which can be prohibitive for long-term studies—and, as many CYPs are involved in endogenous processes, may affect the experimental outcome. The use of 8HUM therefore simplifies the experiment while also delivering an ethical refinement consistent with policies such as the UK-based NC3Rs.

A further important property of 8HUM is the in vivo generation of human metabolites. Metabolite profiles observed in this line were, in many cases, almost identical to profiles in human, and varied markedly from WT mice. This is of particular importance when there are species differences in pharmacologically active or toxic metabolites. For example, during preclinical modeling of efficacy, mouse-specific active metabolites could produce a false positive result, whereas compounds reliant on active metabolite formation for efficacy in humans—such as tafenoquine and primaquine—might not be accurately modeled in WT mice. 8HUM therefore constitutes a more translational model, both for efficacy and safety. Interestingly, our observations with bedaquiline raise the question of whether this compound would ever have been selected for progression had it not produced an active metabolite, and had that metabolite not been present at such high levels in mice. In addition to the 8HUM applications in drug discovery, this also opens the possibility of using 8HUM in the safety assessment of human-specific metabolites.

The 8HUM model contains the enzymes responsible for most of the P450-mediated drug metabolism in humans. However, certain human enzymes are not present, for example, CYP2B6 and CYP2C19, and a number of the murine CYP subfamilies remain, such as Cyp2a, Cyp2b, and Cyp2e. This could potentially confound extrapolation of results in 8HUM to the clinical situation for compounds metabolized by these enzymes. Additional humanizations for these CYPs, or for other drug-metabolizing enzymes or drug transporters, may improve translational accuracy.

CYP3A4 plays a key role in defining the metabolic elimination and metabolite profile of a large number of drugs (6–8). The level of CYP3A4 in the human liver can vary substantially, up to 100-fold (52, 53), and a further application of the model is to create this variability in 8HUM through the activation of CAR or PXR. Although induction of CYP3A4 in 8HUM during routine in vivo drug development work is not practical, this would allow the consequences of such variability on drug efficacy and the potential for DDIs as a consequence of induction to be modeled on a case-by-case basis.

We have shown that the course of infection in 8HUM using three disease models closely matched that of the WT mice. For tuberculosis and Chagas disease, we have demonstrated how 8HUM could be used to bypass issues with high rates of mouse-specific metabolism. Additionally, our observation that the shape of the PK curve in 8HUM is more human-like due to slower elimination suggests three advantages of switching from WT mice to 8HUM in efficacy studies. Specifically, those aimed at characterizing the pharmacokinetic/pharmacodynamic (PK/PD) relationships by dose fractionation (54). First, researchers would not need to spend as much time modifying doses and schedules to match human PK for their PD studies. Second, a broader range of exposures can be tested in 8HUM due to an increase in the upper limit achievable and, third, as differences in compound half-life between mice and human result in divergent effects for a given exposure (55), narrowing of this difference in 8HUM will improve the translational accuracy of the PK/PD assessment (56). Work is scheduled to evaluate additional infection models in 8HUM and in 8HUM/Rag2^−/−^, the immuno-compromised version of this line (25). Further, we anticipate that the utility of the 8HUM and 8HUM/Rag2^−/−^ models will be explored in non-infectious disease drug discovery, with accompanying validation of disease models for cancer, neurodegeneration, cardiovascular disease, and others. In instances where mice have already been genetically modified to better recapitulate human pathophysiology, unless prohibitively complex, these same modifications might in future be made to 8HUM.

Increasingly, treatment of disease involves drug combinations. In areas such as infectious disease and cancer, this is key to suppressing emergence of drug resistance. Combination regimens must be carefully designed to minimize the impact of DDI on therapeutic outcome. Following drug registration, however, co-morbidities are common and often result in patients receiving multiple therapies which have not been developed together, and therefore are more prone to DDI. The propensity of drugs to interact with metabolizing enzymes, as substrates, inhibitors, or inducers, can be investigated using in vitro methodologies, but the extrapolation of these data to patients remains problematic and the impact of these interactions on PK, efficacy and toxicity can only be estimated. To investigate DDIs in vivo requires costly clinical trials which are carried out late in development or post-approval and, by necessity, are limited in scope. Improved preclinical modeling of DDIs using 8HUM would increase the rate at which effective combinations could be progressed toward regulatory approval. In agreement with previous work (24), we found that interactions at the PK level were better represented in 8HUM. Further, we have demonstrated that this can facilitate a preclinical in vivo assessment of the impact of DDI on efficacy, which paves the way for earlier assessment of combination partners and, ultimately, more informed design of combination clinical trials.

In summary, this work demonstrates that the elimination of species differences in drug metabolism through the application of 8HUM, in place of wild-type mice, during preclinical drug discovery can re-focus compound optimization toward human PK. This improves the translation of results while simultaneously increasing the efficiency of development by reducing time and cost. 8HUM therefore has the potential to replace the wild-type mouse in drug discovery.

## Materials and Methods

### Chemicals and Reagents.

All compounds were purchased from Sigma Aldrich/Merck, with the exceptions of bedaquiline, delamanid, efavirenz, linezolid, pretomanid, and ritonavir (Fisher Scientific, Thermo Fisher Scientific). For in vitro studies, compounds were solubilized in dimethyl sulfoxide (DMSO) to 10 mM and entered into storage under nitrogen, at ambient temperature, in the Compound Management facility at the University of Dundee Drug Discovery Unit. Aliquots of these stock solutions were used within 3 mo of solubilization. Human hepatic microsomes (mixed gender pool from 150 individuals) were purchased from Gibco (Thermo Fisher Scientific). In-house preparation of mouse hepatic microsomes was as described in *SI Appendix*. Cryopreserved human and mouse (CD-1) hepatocytes were purchased from Life Technologies (Thermo Fisher Scientific). All LC–MS/MS mobile phase reagents were purchased from Fisher Scientific (Thermo Fisher Scientific). In-house isolation of mouse hepatocytes was as described in *SI Appendix*.

### Western Blotting.

Microsomal samples were adjusted to 1 mg/mL in LDS sample buffer (Life Technologies) for loading of 10 µg total protein into each well and electrophoresis through 10% acrylamide gels for 1 h at 150 V, followed by transfer onto nitrocellulose membranes. Primary antibodies used for immunoblotting were anti-CYP3A4 (458234, BD Biosciences) and anti-GRP78 loading control (ab21685, Abcam). Pooled HLM material was the same as used in microsomal incubations. Loading control is not provided for Fig. 1*B*, as the comparison is between human and murine samples.

### Microsome Incubations for Stability and Metabolite Profiling.

Test compound (0.5 µM final concentration for metabolic stability, 5 µM for metabolite profiling) was combined with microsomes in buffer (0.5 mg/mL 50 mM potassium phosphate, pH 7.4) and the reaction initiated with addition of excess NADPH (final concentration 0.8 mg/mL). Immediately (time zero) then at 3, 6, 9, 15, and 30 min an aliquot (50 µL) of the incubation mixture was removed, mixed with acetonitrile (100 µL), and kept on ice. After all samples were collected, 250 µL of 20% acetonitrile was added to each and the analysis plate was centrifuged for 10 min at 3,000 × g at ambient temperature and analyzed immediately. Internal standard (donepezil, 50 ng/mL) was included in the termination solvent for intrinsic clearance incubations.

### Hepatocyte Incubations for Metabolic Stability.

Cells were incubated in suspension (0.5 million cells/mL), in WME containing CM4000, in 48-well non-collagen coated cell culture plates for 10 min at 37 °C, 5% CO_2_. Reactions were initiated with addition of an equal volume of supplemented WME containing 1 µM test compound, giving a final concentration of 0.5 µM test compound and a final hepatocyte density of 0.25 million cells/mL. Immediately following reaction initiation, and at 3, 6, 9, 15, 30, 45, 60, 90, and 120 min, samples (20 µL) were removed to four volumes of acetonitrile containing internal standard (donepezil, 50 ng/mL) and kept on ice. After all samples were collected, 100 µL of 20% acetonitrile was added to each and the analysis plate was centrifuged for 10 min at 3,000 × g at ambient temperature and analyzed immediately.

### Animal Ethics and Husbandry.

The 8HUM breeding colony was maintained at Charles River Laboratories. Mice were housed in fully flexible isolators with Aspen flake bedding and a conditioned and HEPA-filtered air supply, a 13-h light/11-h dark cycle and ad libitum access to water (filtered and chlorinated with sodium hypochlorite) and food (VRF1 pelleted diet). Temperature and relative humidity were maintained at 21 °C ± 1 °C and 55% ± 10%, respectively. All consumables were irradiated at a minimum of 35 kGy or sterilized by autoclave and/or ethylene dioxide prior to use.

All animal experiments carried out at the University of Dundee were approved by the Ethical Review Committee and performed under the Animals (Scientific Procedures Act) 1986, as amended in 2012, and in accordance with the European Union Directive (2010/63/EU). Animals were inspected regularly by staff trained and experienced in small animal husbandry, with 24-h access to veterinary advice. Mice were maintained in filter top cages (Thoren Mouse Caging System, Thoren) containing Eco-Pure chip7D (Datesand Group) for bedding with ad libitum access to food (RM1; Special Diet Services) and water, and a 12-h light–dark environment. Temperature and relative humidity were maintained between 20 °C and 24 °C, and 45% and 65%, respectively. Experimental design was guided by power calculations (G*Power; https://www.gpower.hhu.de), pilot experiments, and previous experience, and was undertaken in line with the 3Rs principles of replacement, reduction, and refinement (https://www.nc3rs.org.uk).

The tuberculosis infection and efficacy work was carried out at GSK Tres Cantos and, as such, all procedures were performed in accordance with protocols approved by the GSK Institutional Animal Care and Use Committee and met or exceeded the standards of the American Association for the Accreditation of Laboratory Animal Care (AAALAC). All animal studies were ethically reviewed and carried out in accordance with European Directive 2010/63/EU and the GSK Policy on the Care, Welfare, and Treatment of Animals.

### Pharmacokinetic Experiments.

With the exception of quinidine PK, female mice were used in all experiments. Dose levels and schedules were as detailed in *SI Appendix*. Serial blood samples (10 µL) were taken from the tail vein at the timepoints shown, diluted into 9 volumes of Milli-Q water, and stored at −20 °C prior to bioanalysis. Samples from mice infected with *T. cruzi* were snap-frozen in liquid nitrogen and thawed three times before storage at −20 °C until bioanalysis. Samples from mice infected with *M. tuberculosis* (15 µL) were mixed with two volumes of Milli-Q and stored at −70 °C until bioanalysis. All non-compartmental analysis, modeling, and simulation of PK was carried out using Phoenix WinNonlin version 8.3.1.5014 (Certara), as described in *SI Appendix*.

### Infection and Efficacy Studies.

Established models of infection were used for testing compound efficacy against *M. tuberculosis* (41), *L. donovani* (49, 50) and *T. cruzi* (51). Brief summaries of the methodology specific to each model are provided in *SI Appendix*.

### LC–MS/MS Analysis of Samples from In Vitro and In Vivo Studies.

For quantitative bioanalysis, samples from in vitro incubations, PK and *T. cruzi* infection studies were analyzed using an Acquity UPLC system coupled to either a Xevo TQS-Micro (in vitro samples) or Xevo TQ-XS (in vivo samples) mass spectrometer, operated using MassLynx software version 4.2 (Waters). Chromatographic separation was achieved using an Acquity UPLC BEH C18 column, 50 × 2.1 mm, particle size 1.7 µm (Waters, part number 186002350). Samples from *M. tuberculosis* infection studies were analyzed using an Acquity I-Class UPLC (Waters)/QTRAP 6500 mass spectrometer (Sciex). Chromatographic separation was achieved using either a Waters Acquity UPLC BEH C18 column (50 × 2.1 mm, 1.7 µm) for parent compound and Waters Acquity UPLC Premier BEH C18 column (100 × 2.1 mm, 1.7 µm) for metabolites. For qualitative metabolite profiling, samples were analyzed using a Vanquish UHPLC system interfaced with an Exploris 120 mass spectrometer, operated using Xcalibur version 4.4.16.14 (Thermo Fisher Scientific). Chromatography was carried out using a Hypersil Gold C18 column, 50 × 0.21 mm, 1.9 µM particle size (Thermo Scientific, part number 25002-052130). Acquired data were processed in Compound Discoverer v3.2 (Thermo Fisher Scientific). Full analytical methods along with data analysis steps are detailed in *SI Appendix*.

## Supplementary Material

Appendix 01 (PDF)

## Data Availability

All study data are included in the article and/or *SI Appendix*.
