# Supplementary material for: Acceleration of infectious disease drug discovery and development using a humanized model of drug metabolism
Source: Proc Natl Acad Sci U S A. 2024 Feb 5;121(7):e2315069121. doi: 10.1073/pnas.2315069121 (PMC10873626; doi:10.1073/pnas.2315069121)
Supplement: Supplementary file 1 — Appendix 01 (PDF) [file pnas.2315069121.sapp.pdf]

PNAS

Supporting Information for

Acceleration of infectious disease drug discovery and development using a humanized model of drug metabolism

A. Kenneth MacLeod, Kevin-Sebastien Coquelin, Leticia Huertas, Frederick R. C. Simeons, Jennifer Riley, Patricia Casado, Laura Guijarro, Ruth Casanueva, Laura Frame, Erika G. Pinto, Liam Ferguson, Christina Duncan, Nicole Mutter, Yoko Shishikura, Colin J. Henderson, David Cebrian, C. Roland Wolf and Kevin D. Read.

Corresponding author: Kevin D. Read
Email: k.read@dundee.ac.uk

This PDF file includes:

SI Materials and Methods
Table S1
SI References

SI Materials and Methods

Microsome preparation

Mouse hepatic microsomes were prepared by homogenization of freshly-isolated liver in ice-cold SET buffer (0.25 M sucrose, 5 mM EDTA, and 20 mM Tris-HCL, pH 7.4) to make a 10% (w/v) homogenate solution (9 ml SET buffer/1 g liver) using a Polytron PT-MR-2100 homogenizer. Liver homogenates were centrifuged at 2000 rpm (Sorvall RTH-250 rotor) for 10 minutes at 4 °C and the supernatant then centrifuged at 12000 rpm (Sorvall SS-34 rotor) for 20 minutes at 4 °C. The resulting supernatant was centrifuged at 29130 rpm (Sorvall TFT-45.6 rotor) for 90 minutes at 4 °C and the microsomal pellets were resuspended in ice-cold SET buffer for storage at –70 °C.

Hepatocyte isolation

Primary hepatocytes were freshly prepared from male 8HUM using a modified version of Charni-Natan and Goldstein's STAR Protocol*(1)*. Following termination by cervical dislocation and femoral vessel cut for confirmation of death, the inferior vena cava (IVC) was cannulated using a BD Insyte 20-gauge canula (Merck) and the liver perfused using 30 mL Hanks Buffered Saline Solution (HBSS, -Ca^{2+} -Mg^{2+}, no phenol red, Gibco), containing 0.5 mM EDTA and 25 mM HEPES, running at a flow rate of 3 mL/min at approximately 37 ˚C. Subsequently, 10 mL HBSS digestion buffer (Ca2+ & Mg2+, no phenol red, Gibco), including 25 mM HEPES and 270 µL liberase (Merck) running at a flow rate of 3 mL/min and ~37˚C was then perfused. The final perfusion step utilised 30 mL HBSS digestion buffer containing 25 mM HEPES and 20 mg collagenase (Merck) at approximately 37 ˚C. The liver was excised, gall bladder removed and placed in a 10 cm petri dish where it was covered with 10 mL plating medium WME containing CM4000. The liver capsule was gently agitated with a glass rod and forceps to disperse the liver cells. Cells were then poured through a 100 µm nylon strainer (Falcon, Corning, NY, USA) into a 50 mL collection tube and topped up to 50 mL with plating medium at 37 ˚C. All solutions were perfused using a Miniplus peristaltic pump (Gilson, Middleton, WI, USA) and run through a 20 mL Intrafix Safeset bubble trap (Braun, Melsungen, Germany) to prevent potential blocking of vessels during perfusion.

Dose levels and dosing schedules for pharmacokinetic experiments

All compounds were administered as fine suspensions in 0.5% (w/v) hydroxypropyl methylcellulose (HPMC), with the exceptions of efavirenz (0.5% (w/v) HPMC / 0.2% Tween 80 in Milli-Q water) and rifampicin (a solution in 0.1 M potassium phosphate, pH 7.4), at a dose volume of 10 mL/kg. Dose levels were as follows: pretomanid: 5 mg/kg, rifabutin: 3.3 mg/kg, delamanid: 2 mg/kg, bedaquiline: 7.5 mg/kg, quinidine: 10 mg/kg, efavirenz: 40 mg/kg, NCE37-39: 50 mg/kg, rifampicin: 2 mg/kg, clarithromycin: 100 mg/kg, moxifloxacin: 30 mg/kg and ABT: 50 mg/kg. During PK evaluation, NCE_X, NCE_Y and NCE_Z were administered at 50 mg/kg BID for four days. During efficacy study, doses of NCE_X and NCE_Y were reduced to 25 mg/kg BID. For DDI experiments, efavirenz was administered once daily for three days, with the last dose 24 hr prior to administration of pretomanid, and rifampicin was administered once, 24 hr prior to administration of pretomanid. Pre-treatment with SJW was as described previously*(2)*. Briefly, tablets containing extract of SJW (Kira Low Mood Relief SJW extract 450 mg, batch 200270, Artesan Pharma, Luechow, Germany) were crushed using a mortar and pestle and extracted using 100% ethanol. The extract was mixed with PEG400 (final ethanol concentration = 13.9% (v/v)) immediately prior to administration. The SJW dose was 312 mg/kg (assuming complete extraction by ethanol), equivalent to a dose of 6.9 – 9.6 mg/kg of the active component, hyperforin. For pre-treatment of mice used for hepatic microsome preparation, the SJW extract was administered once daily for two days, with the second dose 24 hr prior to liver isolation. For pre-treatment of mice in the clarithromycin PK study, the SJW extract was administered once, 24 hr prior to administration of clarithromycin. This shorter regimen resulted in hepatic induction of CYP3A4 to a level equivalent to the average human level, as assessed by Western blot in comparison to the human liver microsome pool (data not shown).

Tuberculosis infection and efficacy

Briefly, WT and 8HUM mice were housed at ambient temperature (22 ± 2 °C) and 55 ± 15% relative humidity with a 12-hr dark/light cycle and were provided food and water *ad libitum*. Mice were intratracheally infected with approximately 1 x 10^5 colony forming units (CFU) of *M. tuberculosis* H37Rv strain. For evaluation of efficacy, treatment started on day 1 (the day after infection) and regimens were

administered once a day for 8 consecutive days, at 20 mL/kg by oral gavage. In the case of efavirenz, to allow induction of CYP, treatment started 3 days before the other compounds and continued once daily administration for 11 consecutive days. Efavirenz was administered 10-20 minutes before bedaquiline when these compounds were combined. ABT was administered 1 hour before NCE37 when these compounds were combined. Lungs were harvested at the indicated timepoints for analysis of bacterial burden by CFU assay. To determine the CFU counts, lung homogenates were plated in 10% OADC-7H11 medium supplemented with activated charcoal (0.4%) for 18 days at 37 °C. Resulting data were analysed using GraphPad Prism (Dotmatics, Boston, MA, USA).

Leishmania infection

Female balb/c (n = 20) and 8HUM (n = 20) mice were inoculated with *Leishmania donovani* (LV9, WHO designation: MHOM/ET/67/L82) amastigotes prepared from the spleen of a heavily infected donor RAG2-KO mouse via intravenous (*i.v.*) injection into the tail vein. Each mouse was infected with a 0.2 mL bolus of inoculum containing approximately 2 x 10^7 amastigotes. On days 7, 14, 18, 22, 25, 29, 32, 36, 42 and 50 after infection, 2 mice from each strain were terminated and livers and spleens removed. These tissues were weighed, and the parasite load determined microscopically by examining Giemsa-stained smears (Rapi-Diff II, Biotech Sciences Ltd.). The number of amastigotes per 500 liver cells was counted microscopically (10x 100, oil immersion) and the parasite load expressed in Leishman Donovan Units (LDU), calculated by multiplying the mean number of amastigotes per cell by the tissue weight in mg

Chagas infection and efficacy

The infection model utilised a bioluminescent strain of *Trypanosoma cruzi*, CL Brener-Luc, that facilitates *in vivo* imaging to track the course of infection in individual mice through time. Female balb/c and 8HUM mice were infected intraperitoneally (*i.p.*) with approximately 1 x 10^3 trypanosomes taken from the blood of an infected donor RAG2-KO mouse and randomised into experimental groups, each n = 6. On 16 separate occasions from day 0 (infection) to day 154 (termination), mice were anaesthetised using isoflurane for *in vivo* imaging of trypanosome bioluminescence intensity using an IVIS Lumina XR system (Caliper Life Sciences, Perkin Elmer, Hopkinton, MA, USA). Beginning on day 135 post-infection, experimental groups were treated for 20 days as follows. Group 1 was treated twice daily with vehicle (1% (w/v) methylcellulose (MC)). Group 2, acting as positive control, was treated once daily with 100 mg/kg benznidazole in 5% DMSO, 95% HPMC (0.5% (w/v) in Milli-Q water). Groups 3, 4 and 5 were treated twice daily with NCE_X, NCE_Y and NCE_Z, respectively. All three of these compounds were administered in 1% MC, with NCE_X and NCE_Y administered at 25 mg/kg and NCE_Z at 50 mg/kg. To facilitate the detection of residual infection after treatment, mice were immunosuppressed with 200 mg/kg cyclophosphamide (Sigma) by *i.p.* injection ten days after end of treatment, at three day intervals, for a maximum of three doses. Fourteen days after the last dose of cyclophosphamide, mice were assessed by both *in vivo* and *ex vivo* imaging. Mice that were bioluminescence negative by both were scored as “cured”.

LC-MS/MS analysis of samples from *in vitro* incubations, PK and *T. cruzi* infection studies

For *in vitro* samples, the column was held at 45 °C, mobile phase A was 0.01% formic acid in Milli-Q water, mobile phase B was 0.01% formic acid in LC-MS grade methanol and the gradient programme was as follows: 0.0 – 0.3 min: 5% B, 0.3 – 1.3 min: 5 to 95% B, 1.3 – 1.8 min: 95% B and 1.8 – 1.81 min: 95 – 5% B. For PK sample analysis, the column was held at 40 °C, mobile phase A was 0.01% formic acid in LCMS grade water, mobile phase B was 0.01% formic acid in LCMS grade acetonitrile and the gradient programme was as follows: 0.0 – 0.5 min: 5% B, 0.5 – 2.0 min: 5 to 95% B, 2.0 to 2.5 min: 95% B and 2.5 – 3.4 min: 95 – 5% B. Flow rate was 0.6 mL/min for all methods. The mass spectrometer was operated with electrospray ionisation in positive mode, with capillary voltage of 0.8 kV, desolvation temperature of 500 °C (in vitro samples) or 600 °C (PK samples), desolvation gas flow at 1000 L/hr, cone gas flow at 150 L/hr and source temperature at 150 °C. Multiple reaction monitoring (MRM) transitions, cone voltages and collision energies were optimised for each compound using QuanOptimise software.

LC-MS/MS analysis of samples from *M. tuberculosis* infection studies

Samples (10 µL) were combined with 190 µL of acetonitrile:methanol (80:20) containing internal standard (midazolam) and transferred to a 0.45 µm filter plate. Samples were vortexed for 30 seconds and filtered with a vacuum of -20 psi. Samples were transferred to a 0.22 µm filter plate and filtered a second time. During LC-MS/MS analysis, the mass spectrometer was operated in positive mode using transitions of 557.171 / 58.1 m/z for bedaquiline, 541.132 / 480.1 m/z for NDM-bedaquiline, 360.039 / 175.054 m/z for pretomanid and 326.1 / 291 m/z for midazolam

Intrinsic clearance data analysis

Raw LC-MS/MS data were exported to XLfit (IDBS, Woking, UK) for calculation of exponential decay rate constant (k) from the ratio of peak area of test compound to internal standard at each timepoint. For microsomal incubation data, the rate of intrinsic clearance (CL_{int}) of each test compound was then calculated using the following formula:

CL_{int} (mL/min/g liver) = k x V x microsomal protein yield

Where V (mL/mg protein) is the incubation volume/mg protein added and microsomal protein yield is taken as 52.5 mg protein / g liver *(3)*. Verapamil was used as a positive control to confirm acceptable assay performance. For hepatocyte incubation data, the following formula was used:

CL_{int} (mL/min/g liver) = k/V x hepatocellularity scaling factor

Where V is million cells per mL and the hepatocellularity scaling factor of 120 x 10^6 cells / g of liver for both human and mouse was applied *(3)*. Verapamil, 7-ethoxycoumarin, 7-hydroxycoumarin and phthalazine were used as positive controls to confirm acceptable assay performance. For all intrinsic clearance measurements, lower and uppers limits of quantitation were 0.5 and 50 mL/min/g liver, respectively. Values were transferred to RStudio v1.3.1093 (Posit, Boston, MA, USA) for generation of scatter plots and heatmaps using the ggplot2 *(4)* package.

Quantitative bioanalysis of PK samples

On the day of sample analysis, calibration standards (CS) and quality controls (QC) were prepared from separate aliquots of test compound certified ≥ 99% pure as supplied or verified to the same level in-house by LC-UV. Compounds were dissolved in DMSO and spiked into control matrix (blank blood diluted to the same level in Milli-Q water as used during PK sampling) prior to extraction with three volumes of acetonitrile containing IS (donepezil at 5 ng/mL), in parallel with PK samples. Concentration of drug in samples was determined by interpolation onto the linear regression model (weighting: 1/x) fitted to CSs using TargetLynx (Waters). Acceptance criteria of the bioanalytical method included CS accuracy of ± 20% of nominal (theoretical) concentration across the range of interest. Lower limit of quantification (LLoQ) was determined as the analyte response with ≥ three times the analyte response of single blanks (extracted blood containing IS but no test compound). QCs at low, medium, and high concentration levels were injected throughout the analytical run, with an acceptance criterion of 66% of injected samples being within ± 20% of nominal (theoretical) concentrations. All single and double blanks (extracted blood with no IS or test compound) were verified as free of interference at the retention times of the IS and test compound. Sample carryover was determined in a blank injection immediately following the injection of the top CS. Carryover was deemed accepted when the test compound response in the blank was < 1% of that in the top calibration sample.

Metabolite profiling

Blood samples were analysed using a Vanquish UHPLC system interfaced with an Exploris 120 mass spectrometer, operated using Xcalibur version 4.4.16.14 (Thermo Fisher Scientific). Chromatography was carried out using a Hypersil Gold C18 column, 50 x 0.21 mm, 1.9 µM particle size (Thermo Scientific, part number 25002-052130), held at 40 °C. Mobile phase A was 0.01% formic acid in LCMS grade water, mobile phase B was 0.01% formic acid in LCMS grade acetonitrile and the gradient programme was as follows: 0.0 – 7.0 min: ramp from 2% B to between 35 and 95% B, depending on retention time of the parent compound, 7.0 – 8.0 min: ramp to 95% B, 8.0 – 8.05 min: 95 to 2% B and 8.05 – 9.0 min: hold at 2% B. The flow rate was 0.5 mL/min. The mass spectrometer was operated with

electrospray ionisation in positive mode, with a spray voltage of 3500 V, ion transfer tube at 320 °C, vaporizer at 375 °C and sheath, aux and sweep gases of 60, 15 and 1 arbitrary units, respectively. Full scan was set at a resolution of 30,000 with scan range of 120-1,200 m/z and RF lens of 70%. Top four ions were selected for data-dependent MS2 acquisition, with an isolation window of 1.5 m/z, resolution of 15,000, and normalised collision energies of 15, 30 and 45%. Expected peak widths were set to 4 sec and dynamic exclusion and maximum injection time mode were set to automatic. Acquired data were processed in Compound Discoverer v3.2 (Thermo Fisher Scientific). Background subtraction was applied using blank matrix extracts and putative metabolites were annotated based on mass accuracy against nominal (± 3 ppm tolerance), isotope pattern and feasibility of biotransformation, with additional monitoring of peak shape and MS intensity relative to sampling time. In some cases, structural elucidation was carried out through interpretation of fragmentation spectra, and in consideration of (*i*) *in silico* predictions generated using MetaSite (Molecular Discovery Ltd., Borehamwood, UK) and ADMET Predictor (Simulations Plus, Lancaster, CA, USA), (*ii*) FDA and EMA review documentation and (*iii*) published studies. Peak area tables for putative metabolites were exported for further processing in Microsoft Word. For *in vitro* data, peak areas at the final timepoint of the incubation – 30 minutes – were normalisation to the peak area of the respective parent compound at time zero, with conversion to a percentage value. *In vitro* metabolite relative abundance values, and metabolite peak areas from PK samples, were transferred to RStudio for plotting and principal components analysis (PCA) using the ggplot2*(4)* and factoextra*(5)* packages, respectively.

***In silico* modelling of PK data**

PK parameters were obtained by non-compartmental analysis of blood concentration *vs.* time data using Phoenix WinNonlin version 8.3.1.5014 (Certara, Princeton, NJ, USA). For compartmental modelling and simulation, drug concentrations in whole blood from WT and 8HUM PK studies were converted to plasma levels using predicted mouse blood:plasma values of 0.788 for pretomanid, 0.749 for delamanid and 0.956 for rifabutin, as determined using ADMET Predictor (v10.4, Simulation Plus, Lancaster, CA, USA). These values were used to generate models describing mouse PK in Phoenix WinNonlin. For the equivalent human models, plasma concentration *vs.* time data for orally-administered pretomanid *(6)* (250 mg single dose) and rifabutin *(7)* (450 mg single dose) was extracted from published figures using WebPlotDigitizer (https://automeris.io/WebPlotDigitizer). One-compartment models were used for all mouse and human PK data, with the exception of human rifabutin, which was better-described with two compartments. As PK data was unavailable for delamanid, a one-compartment model was generated using reported values for apparent volume of distribution (V/F) of 11.9 L/kg and oral clearance (CL/F) of 9.12 mL/min/kg, following administration of the recommended 100 mg BID dose *(8)*. The predicted exposure from the resulting profile was in reasonable agreement with reported values *(9)*. These models were used to simulate steady-state conditions using the nonparametric superpositioning approach. The human model for pretomanid was adjusted to reflect the recommended dose level of 200 mg QD. Auto-induction of rifabutin metabolism has been reported in human *(7)* but as this is weak and there is no auto-induction in mouse *(10)*, it was not factored into the repeat dose rifabutin simulations here.

Table S1. Compounds identified for study. Classifications as substrate, inhibitor and/or inducer are based on FDA and EMA documents indexed by PharmaPendium and the University of Washington DIDB.

Compound	Indication	CYP interaction		
		Substrate	Inhibitor	Inducer
Amoxicillin	TB		2C19	
Bedaquiline	TB	3A4		
Ciprofloxacin	TB		1A2, 2D6	
Clarithromycin	TB	3A4		
Delamanid	TB	3A4		1A2
Linezolid	TB	3A4		
Pretomanid	TB	3A4		
Rifabutin	TB	3A4		
Rifampicin	TB			3A4, 2C9, 2C19
Rifapentine	TB			2C8, 2C9
Fluconazole	Chagas		2C9, 2C19	2C9, 2C19
Posaconazole	Chagas	3A4	3A4	
Artemether	Malaria		3A4	
Erythromycin	Malaria		3A4	
Halofantrine	Malaria	3A4		
Mefloquine	Malaria	3A4		
Oleandomycin	Malaria		3A4	
Proguanil	Malaria	2C19		
Quinidine	Malaria		2D6	
Amprenavir	HIV		3A4	
Darunavir	HIV		3A4	
Efavirenz	HIV	2B6		3A4
Etravirine	HIV	2C9		
Indinavir	HIV	3C8, 2C9	3A4	
Nelfinavir	HIV		3A4	
Ritonavir	HIV		3A4	
Tenofovir	HIV	1A2		

SI References

1. M. Charni-Natan, I. Goldstein, Protocol for Primary Mouse Hepatocyte Isolation. *STAR Protoc.* **1**, 100086 (2020)
2. C. J. Henderson, Y. Kapelyukh, N. Scheer, A. Rode, A. W. McLaren, A. K. MacLeod, D. Lin, J. Wright, L. A. Stanley, C. R. Wolf, An extensively humanized mouse model to predict pathways of drug disposition and drug/Drug interactions, and to facilitate design of clinical trials. *Drug Metab. Dispos.* **47**, 601–615 (2019)
3. T. Iwatsubo, N. Hirota, T. Ooie, H. Suzuki, N. Shimada, K. Chiba, T. Ishizaki, C. E. Green, C. A. Tyson, Y. Sugiyama, Prediction of in vivo drug metabolism in the human liver from in vitro metabolism data. *Pharmacol. Ther.* **73**, 147–171 (1997)
4. H. Wickham, *ggplot2: Elegant Graphics for Data Analysis* (Springer-Verlag New York, 2016)
5. A. Kassambara, F. Mundt, Factoextra: Extract and Visualize the Results of Multivariate Data Analyses (2020) (available at https://cran.r-project.org/package=factoextra).
6. A. M. Ginsberg, M. W. Laurenzi, D. J. Rouse, K. D. Whitney, M. K. Spigelman, Safety, tolerability, and pharmacokinetics of PA-824 in healthy subjects. *Antimicrob. Agents Chemother.* **53**, 3720–3725 (2009)
7. M. S. Benedetti, C. Efthymiopoulos, D. Sassella, E. Moro, M. Repetto, Autoinduction of rifabutin metabolism in man. *Xenobiotica* **20**, 1113–1119 (1990)
8. L. Tanneau, M. O. Karlsson, A. H. Diacon, J. Shenje, J. De Los Rios, L. Wiesner, C. M. Upton, K. E. Dooley, G. Maartens, E. M. Svensson, Population Pharmacokinetics of Delamanid and its Main Metabolite DM-6705 in Drug-Resistant Tuberculosis Patients Receiving Delamanid Alone or Coadministered with Bedaquiline. *Clin. Pharmacokinet.* **61**, 1177–1185 (2022)
9. M. T. Gler, V. Skripconoka, E. Sanchez-Garavito, H. Xiao, J. L. Cabrera-rivero, D. E. Vargas-vasquez, M. Gao, M. Awad, S. Park, T. S. Shim, G. Y. Suh, M. Danilovits, H. Ogata, A. Kurve, J. Chang, K. Suzuki, T. Tupasi, W.-J. Kon, B. Seaworth, L. J. Geiter, C. D. Wells, Delamanid for multidrug-resistant pulmonary tuberculosis. *N. Engl. J. Med.* **7**, 2151–2160 (2012)
10. M. S. Benedetti, E. Pianezzola, G. Brianceschi, D. Jabes, C. D. Bruna, M. Rocchetti, I. Poggesi, An investigation of the pharmacokinetics and autoinduction of rifabuting metabolism in mice treated with 10 mg/kg/day six times a week for 8 weeks. *J. Antimicrob. Chemother.* **36**, 247–251 (1995)
